# Auditory-Visual Object Recognition Time Suggests Specific Processing for Animal Sounds

**DOI:** 10.1371/journal.pone.0005256

**Published:** 2009-04-22

**Authors:** Clara Suied, Isabelle Viaud-Delmon

**Affiliations:** Institut de Recherche et de Coordination Acoustique / Musique (IRCAM) - Centre National de la Recherche Scientifique (CNRS), UMR 9912, Paris, France; Istituto di Neurofisiologia, Italy

## Abstract

**Background:**

Recognizing an object requires binding together several cues, which may be distributed across different sensory modalities, and ignoring competing information originating from other objects. In addition, knowledge of the semantic category of an object is fundamental to determine how we should react to it. Here we investigate the role of semantic categories in the processing of auditory-visual objects.

**Methodology/Findings:**

We used an auditory-visual object-recognition task (go/no-go paradigm). We compared recognition times for two categories: a biologically relevant one (animals) and a non-biologically relevant one (means of transport). Participants were asked to react as fast as possible to target objects, presented in the visual and/or the auditory modality, and to withhold their response for distractor objects. A first main finding was that, when participants were presented with unimodal or bimodal congruent stimuli (an image and a sound from the same object), similar reaction times were observed for all object categories. Thus, there was no advantage in the speed of recognition for biologically relevant compared to non-biologically relevant objects. A second finding was that, in the presence of a biologically relevant auditory distractor, the processing of a target object was slowed down, whether or not it was itself biologically relevant. It seems impossible to effectively ignore an animal sound, even when it is irrelevant to the task.

**Conclusions/Significance:**

These results suggest a specific and mandatory processing of animal sounds, possibly due to phylogenetic memory and consistent with the idea that hearing is particularly efficient as an alerting sense. They also highlight the importance of taking into account the auditory modality when investigating the way object concepts of biologically relevant categories are stored and retrieved.

## Introduction

Representing and processing object concepts is a major challenge for human perception and cognition. We can easily categorize and differentiate different classes of objects: for instance, such structurally complex and disparate things as penguins, camels, dogs, and sparrows are recognized as animals seemingly without any effort, and in any case very fast [1]. Another important observation is our use of multisensory information to recognize objects in our environment. To recognize an object, we must usually bind together sensory cues coming from the object through different sensory modalities, while at the same time ignoring sensory information originating from competing objects. In this paper we investigate the role of semantic categories (animal vs. means of transport) in selective processing of competing sensory information.

Increasing evidence suggests that different categories of objects are not processed in the same way. Ones of the first intriguing set of results came from neuropsychological studies describing patients with category-specific semantic impairment. Some patients exhibited relatively better knowledge of animate categories than inanimate objects [2]; and the reverse pattern has also been described [3]. Different models of the semantic system have been proposed to account for the neuropsychological findings (for different views and models, see [4]–[7]). Importantly, only few of them integrate the role of the auditory information in the access to stored knowledge (although see e.g. [8]–[9] for studies on auditory agnosia). The majority of neuropsychological studies were mainly concerned with visual recognition, and the interplay between different sensory modalities was rarely considered in the description of category-specific impairments of object recognition.

Again in the unimodal case, brain imaging studies in healthy participants have shown that the representation of specific semantic categories (including faces and voices) differ from each other (e.g. [10] for the visual modality; [11]–[14] for the auditory modality). Overall in these studies, the comparison of biologically relevant (faces, animals cries, voices) and non biologically relevant (tools, means of transport) stimuli have exhibited the existence of distinct cortical pathways for processing these different categories. In contrast, the only available behavioural results revealed no advantage in the speed of processing when comparing visual stimuli of animals and means of transport [15], or auditory stimuli of living and man-made objects [13].

However, unimodal situations may be considered as ones of sensory deprivation, compared to our natural environment where perception of an object is commonly a multisensory process. Semantic congruence can play a role in the integration of auditory and visual information (review in [16]). A recent study investigated the effect of object categories in auditory-visual integration [17]. Using fMRI and identification rates, they showed specific multisensory associations for biologically relevant stimuli (human voice and faces), compared to non-biologically relevant stimuli (cell phones). A first question addressed in the present study is to measure the processing time for identification of a biologically relevant category compared with to a non-biologically relevant one, when object information is presented in several sensory modalities (auditory and visual).

Another way of addressing the processing of semantic categories is to consider their potential role as distractors, that is, as competing objects irrelevant to the target task. In an auditory-visual object recognition experiment, we previously observed that reaction times were slower with a visual target and an auditory distractor than for a visual target alone [18]. The reverse was not true: there was no “interference effect” caused by visual distractors when the task was to recognize auditory targets. This suggests a possible asymmetry in the attentional filtering of auditory and visual modalities. However, the two objects we used belonged to different categories: an animal as a distractor, and an inanimate object as a target. In the present study, we investigated whether the efficiency of attentional filtering depends on the semantic category of the target or the distractor.

The three experiments presented here share a similar procedure. Participants had to recognize as fast as possible (go/no-go paradigm) a predefined target, belonging to one of two superordinate categories. One category was biologically relevant (animals) and the other was non-biologically relevant (means of transport). In the first two experiments, we compared the respective role of the target and distractors' nature. In Experiment I, the target was a means of transport and the distractors were animals. In the Experiment II, the target was an animal and the distractors were means of transport. Finally, in Experiment III, we studied a possible specific alerting role of animal sounds suggested by the results of the first two experiments: the target *and* the distractors were animals.

## Methods

### Participants

Twelve participants (all right-handed; 6 women; mean age±standard deviation = 24.8±4.5 years) participated in Experiments I and II in a counterbalanced order (hereafter, group 1 designates the group of participants who performed first the Experiment I; group 2 designates the second group of participants who performed first the Experiment II). Twelve other participants (all but 2 right-handed; 5 women; mean age±standard deviation = 33.3±11.6 years) participated in Experiment III. All were naïve with respect to the purpose of the experiment. None of them reported having hearing problems, and all reported normal or corrected-to-normal vision. The study was carried out in accordance with the Declaration of Helsinki and following the rules of the COPé of the CNRS (Comité Opérationnel pour l'éthique). All participants provided written informed consent to participate in the study.

### Apparatus

**Figure 1:**
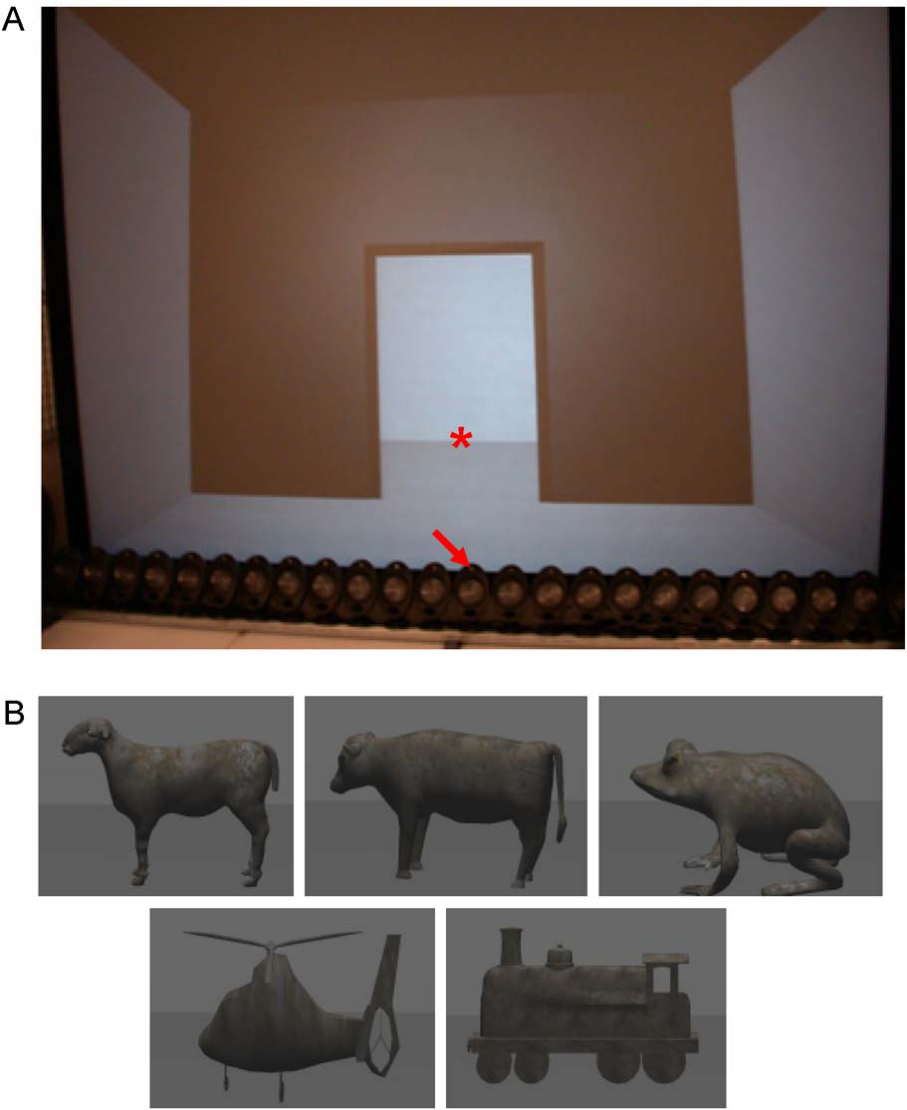
Setup and visual stimuli used in the experiments. Panel A: the setup used in the experiment is composed of a large screen and loudspeakers. Stimuli are projected on the visual background representing a door. The asterisk indicates the location for the visual stimulus; the arrow indicates the loudspeaker used for the auditory stimuli. Panel B: screenshots of the visual stimuli (the sheep, the cow, the frog, the plane and the train).

The experiment took place in an acoustically damped and sound proof recording studio with the light switched off. The visual scene was presented on a 300×225 cm^2^ stereoscopic passive screen (corresponding to 90°×74° at a viewing distance of 1.5 m) and was projected with two F2 SXGA+ ProjectionDesign projectors. Participants wore polarized glasses. Auditory stimuli were presented via a KEF loudspeaker situated at 0° in azimuth, straight ahead at a distance of 1.5 m, aligned with the visual stimuli (see Figure 1). During the experiment, a serial response box (Cedrus Corporation, model RB-730) was used to record participants' response time and accuracy.

### Stimuli

Stimuli representing means of transport and animals were used, either visual and/or auditory. The duration of each stimulus was 500 ms. For Experiment I, the target was a means of transport (a train) and the two distractors were animals (a frog and a cow). For Experiment II, the target was an animal (a frog) and the two distractors were means of transport (a train and a plane). For Experiment III, the target and the distractors were animals (target: frog; distractors: sheep and cow).

#### Images

3D models were all obtained from a database designed for this experiment by INRIA. All the images were adjusted to the same size in all cardinal dimensions. They subtended 8° in the vertical angle and 11° in the horizontal angle. In addition, the same texture, colour, and the same illumination parameters were applied to all visual stimuli. The visual stimulus was embedded in a virtual environment representing a room; objects appeared behind a door situated in the centre of this room (see Figure 1).

#### Sounds

Auditory stimuli were complex sounds (16 bit; 44100 Hz digitization), all obtained from the Hollywood Edge database. They were modified to be 500 ms in duration. They were all equalized in loudness prior to the experiment (to avoid any RT differences due to loudness differences; see [19]–[20]). The train sound was presented at 69 dB SPL, the frog sound was presented at 64 dB SPL the cow sound was presented at 70 dB SPL, the plane sound was presented at 65 dB SPL, and the sheep sound was presented at 67 dB SPL. The sounds used during the experiment were correctly identified by six listeners in a pilot study (with the sounds presented via loudspeakers).

### Procedure

The go/no-go task we used is described in details in [18]. Briefly, participants were asked to press the response button with their right index if the target was present, either in the visual and/or auditory modality, and to withhold any response to distractor stimuli.

Unimodal stimulus conditions could be visual or auditory. Bimodal conditions could be either semantically congruent (an image and a sound belonging to the same object) or incongruent (an image and a sound belonging to different objects). Incongruent conditions included both task-relevant (target) and task-irrelevant (distractor) information. All stimulus conditions are detailed in Table 1.

**Table 1:** Reaction times and accuracy for each condition, defined as a possible combination of target (T) and/or distractor (D) objects in the visual and/or auditory modality.

Condition	Exp I T = transp D = anim		Exp II T = anim D = transp		Exp III T = anim D = anim	
	RT (ms)	% Misses	RT (ms)	% Misses	RT (ms)	% Misses
**Targets (go)**						
**Unimodal**						
A+ (auditory target)	431±20	6.4±1.1	439±22	3.7±1.0	435±16	0.9±0.5
V+ (visual target)	387±13	4.2±1.2	391±14	4.9±1.1	405±11	0.3±0.2
**Bimodal congruent**						
A+V+ (auditory and visual targets)	347±14	4.8±1.3	351±15	4.0±0.9	351±11	0.7±0.4
**Bimodal incongruent**						
A−V+_1_ (visual target and auditory distractor 1)	406±13	2.7±0.8	376±15	1.0±0.5	419±13	0.7±0.6
A−V+_2_ (visual target and auditory distractor 2)	415±16	1.8±0.4	382±15	2.1±0.5	428±9	0.6±0.5
A+V−_1_ (auditory target and visual distractor 1)	441±27	3.3±0.9	437±21	2.7±0.6	421±21	0.4±0.3
A+V−_2_ (auditory target and visual distractor 2)	416±24	2.7±0.8	454±21	3.0±0.7	417±18	0.7±0.6
	**Exp I T = transp D = anim**		**Exp II T = anim D = transp**		**Exp III T = anim D = anim**	
		**% FA**		**% FA**		**% FA**
**Non-targets (no-go)**						
**Unimodal**						
A− (auditory distractors)		3.6±1.2		5.2±1.5		6.3±2.8
V− (visual distractors)		4.2±1.4		5.2±1.9		6.3±1.7
**Bimodal congruent**						
A−V−c (auditory and visual distractors congruent)		10.4±2.6		10.4±1.8		14.1±3.9
**Bimodal incongruent**						
A−V−i (auditory and visual distractors incongruent)		19.3±2.1		10.4±3.0		14.6±2.5

Target and distractor objects could belong to two different categories: means of transport (designated by transp) or animals (designated by anim). In Experiment I, the target is a means of transport (train) and the distractors are two animals (frog and cow). In Experiment II, the target is an animal (frog) and the distractors are two means of transport (train and plane). In Experiment III, the target is an animal (frog) and the distractors are also animals (sheep and cow). RTs±standard error of the mean (SEM) and percentage of misses±SEM are detailed for each go condition, as the percentage of false alarms (FA)±SEM for each no-go condition. RTs were first transformed to a log scale and then averaged across all participants. The log scale is converted back to ms for clarity. For the non-targets (i.e. no-go) conditions, the percentage of false alarms were averaged across the two distractors 1 and 2 for each condition (A−, V−, A−V−c, and A−V−i) to have a sufficient number of presentations for each percentage.

The five go conditions (A+, V+, A+V+, A−V+, A+V−) were presented 56 times each in total per participant. For the two bimodal incongruent conditions (A−V+ and A+V−), there were two different pairings of the sound and the image, one with each distractor (28 repetitions for each pairing). The four no-go conditions (A−, V−, A−V−_c_, A−V−_i_) were presented 16 times each in total (8 times for each distractor). The entire experiment for each participant consisted of 344 stimuli of which 280 (i.e., 81%) were task-relevant stimuli (go responses).

These stimuli were presented on four separate blocks of trials. Participants performed practice trials until they were comfortable with the task. The inter stimulus interval (ISI) was varied randomly between 1.5 and 3 seconds. The order of the stimuli presentation was pseudo-randomized. The entire experimental session lasted about 30 min.

### Statistical analyses

All different statistical analyses are also described in details in [18]. Responses were first analysed to remove error trials (RTs less than 100 ms and RTs greater than 1000 ms). Percentage of false alarms and percentage of misses were analyzed by one-way nonparametric repeated measures analyses of variance (ANOVA; the Friedman test). *p*<.05 was considered to be statistically significant.

Each RTs value was transformed to its natural logarithm (ln), before averaging ln(RT) for each condition. To identify between-condition differences in mean ln(RTs), a repeated-measures ANOVA was conducted with Condition as a within-subjects factor (A+, V+, A+V+, A−V+ and A+V−) (and, for Experiments I and II, with Group (group 1, group 2) as a between-subjects factor). A Kolmogorov-Smirnov test was performed to check for the normality of the distribution of residuals of the ANOVA. For this analysis, we pooled together the results for all conditions in order to increase the power of the statistical test. Finally, to account for violations of the sphericity assumption, *p*-values were adjusted using the Huynh-Feldt correction. *p*<.05 was considered to be statistically significant. In addition, when a significant effect of Condition was found, four planned comparisons were performed to study: (1) RT bimodal facilitation (A+V+ compared to A+ and V+ considered together); (2) the comparison between both unimodal conditions (A+ compared to V+); (3 and 4) interference effects (comparison of V+ with A−V+ and A+ with A+V−). Since these planned comparisons were non-orthogonal, a p-value of .0125 was considered as statistically significant (.0125 = .05 / 4, where 4 is the number of planned comparisons and .05 the alpha-level). Finally, we also checked for potential differences between distractors, either in the visual or in the auditory modality, to be sure that potential interference effects observed were not due only to one of the two distractors. We considered seven different conditions (A+, V+, A+V+, A−V+_1_, A−V+_2_, A+V−_1_, A+V−_2_), where the subscript 1 and 2 represent the two different distractors. Four planned comparisons were performed (still with a p-value of .0125 considered significant, because these comparisons were non orthogonal) to study: (1 and 2) the interference effect for auditory distractors (V+ compared with A−V+_1_ and V+ compared with A−V+_2_); (3 and 4) the interference effect for visual distractors (A+ compared with A+V−_1_ and A+ compared with A+V−_2_).

To determine if the bimodal semantically congruent stimuli (A+V+) resulted in responses that were faster than predicted on the basis of both unimodal stimuli (A+ and V+) processed independently, RTs distributions were estimated against the race model prediction [21]–[22]. The race model is a separate activation model; it assumes that the auditory and the visual components of the bimodal stimulus are processed independently, so it predicts shorter RTs for bimodal stimulus purely on the basis of statistical facilitation. On the contrary, the coactivation model postulates a convergence between the two components of the bimodal stimulus (with no hypothesis on the locus of this convergence). We used the algorithm described in [23] to test the race model. Here, we compared the A+V+ condition to both V+ and A+ conditions.

## Results

### Experiment I: means of transport as target, animals as distractors

All values of RTs, false alarms and misses (±SEM) are presented in Table 1.

#### Accuracy

Nonparametric repeated-measures ANOVA (Friedman's test) revealed a significant effect of Condition (V−, A−, A−V−c, and A−V−i) on percentage of FA (χ^2^(3) = 25.43; *p*<.0001). The percentage of FA was higher with a bimodal stimulus than with a unimodal one (see Table 1), thus revealing a small speed-accuracy trade-off (RTs were faster for bimodal semantically congruent stimuli than for unimodal stimuli; see *Reaction Times*). In addition, the percentage of FA was higher for a bimodal semantically incongruent stimulus than with a bimodal semantically congruent one (see Table 1).

The Friedman's test revealed no significant effect of Condition (A+, V+, A+V+, A−V+ and A+V−) on the percentage of Misses (χ^2^(4) = 7.08; *p* = .13).

#### Reaction Times

No anticipations were found and only 2 RTs out of the 3360 responses were longer than 1000 ms and had to be discarded. A Kolmogorov-Smirnov test was performed on the distribution of the residuals of the ANOVA and revealed that this distribution was not different from a normal distribution (d = 0.11; N = 60; *p*>.1). This result validates the log-transformation and shows that the original distribution of RTs was indeed lognormal.

The ANOVA comparing ln(RTs) revealed a significant main effect of Condition (*F*(4, 40) = 34.75; *ε* = 0.4; *p*<.0001). No significant effects of the Group (*F*(1, 10) = 1.09; *p* = .3) nor of the interaction between Group and Condition (*F*(4, 40) = 2.14; *p* = .09) were found.

Planned comparisons revealed that: (1) RTs were significantly faster for the bimodal condition (A+V+) than for both unimodal conditions (V+ and A+) considered together (*t*(4) = 18.2, *p*<.0001); (2) RTs to unimodal visual stimuli were faster than RTs to unimodal auditory stimuli (*t*(4) = 7.1, *p*<.0001); (3) RTs to A−V+ were significantly slower than RTs to V+ (*t*(4) = 3.6, *p*<.005), thus revealing an interference effect when the target was visual and the distractor was in the auditory modality; (4) RTs to A+V− were equivalent to RTs in the A+ condition (*t*(4) = 0.5, *p* = .6): when the target was auditory, the visual distractor did not influence RTs. These results are represented in Figure 3 (panel A).

Finally, no differences were observed between the two distractors. There was always an interference effect when the distractor was auditory, either for a frog sound or for a cow sound: (1) V+ was significantly different from A−V+_1_ (*t*(4) = 3.0, *p*<.01) and was also (2) significantly different from A−V+_2_ (*t*(4) = 3.0, *p*<.005). There was never any interference effect when the distractor was visual, either for an image of a frog or for an image of a cow: (3) A+ was similar to A+V−_1_ (*t*(4) = 0.6, *p* = .6) (4) and was also similar to A+V−_2_ (*t*(4) = 1.9, *p* = .09).

#### Test of the Race Model

The race model that compared A+V+ to V+ and A+ was significantly violated (*p*<.01) for all percentiles in the lower part of the RTs, i.e. from the .025^th^ to .625^th^. Thus, the race model could not fully explain the pattern of RTs observed for the bimodal stimulus. There was a coactivation – as opposed to separate activations – of the auditory and the visual modalities during the bimodal conditions. This result supports integration accounts for faster RTs in bimodal than in unimodal conditions of presentation.

### Experiment II: animal as target, means of transport as distractors

#### Accuracy

The Friedman's test revealed a significant effect of Condition (V−, A−, A−V−c, and A−V−i) on the percentage of FA (χ^2^(3) = 8.06; *p*<.05). It was higher with a bimodal stimulus than with a unimodal one; there was no difference between the bimodal incongruent condition and the bimodal congruent one (see Table 1).

The Friedman's test revealed a significant effect of Condition (A+, V+, A+V+, A−V+ and A+V−) on the percentage of Misses (χ^2^(4) = 10.59; *p*<.05), which was due to a fewer number of misses in the incongruent conditions compared to the unimodal and bimodal congruent conditions.

#### Reaction Times

No anticipations were found and only 2 RTs out were longer than 1000 ms. The distribution of the residuals of the ANOVA was not different from a normal distribution (d = 0.07; N = 60; *p*>.1).

The ANOVA comparing ln(RTs) revealed a significant main effect of Condition (*F*(4, 40) = 59.50; *ε* = .8; *p*<.0001), and no significant main effect of the Group (*F*(1, 10) = 0.04; *p* = .8). A significant interaction between Group and Condition (*F*(4, 40) = 3.07; *ε* = 0.8; *p*<.05) was found.

The planned comparisons performed to explore the main effect of Condition revealed that: (1) RTs were significantly faster for the bimodal condition (A+V+) than for the unimodal conditions (V+ and A+) considered together (*t*(4) = 19.8, *p*<.0001); (2) RTs to unimodal visual stimuli were faster than RTs to unimodal auditory stimuli (*t*(4) = 5.6, *p*<.001); (3) RTs to A−V+ were similar to RTs to V+ (*t*(4) = 2.3, *p* = .04), thus revealing *no interference effect* when the animal target was visual and the means of transport were distractor in the auditory modality; (4) RTs to A+V− were equivalent to RTs to the A+ condition (*t*(4) = 0.3, *p* = .7). These results are represented in Figure 3 (panel B).

As in Experiment I, there were no differences between both distractors: (1) V+ was similar to A−V+_1_ (*t*(4) = 2.3, *p* = .04) and (2) to A−V+_2_ (*t*(4) = 1.9, *p* = .09). (3) A+ was similar to A+V−_1_ (*t*(4) = 0.9, *p* = .3) (4) and to A+V−_2_ (*t*(4) = 1.3, *p* = .2).

There seems to be a slight influence of the Group depending on the Condition. Overall, participants from group 1 tended to be faster in general (although there was no significant effect of the factor Group for both experiments 1 and 2; see also results of Experiment 1). We thus analysed in more details ln(RTs) in each condition (A+, V+, A+V+, A−V+ , and A+V−) for each group separately to ensure that the main effect of interest in this study is not only a bias due to this interaction. Importantly, the pattern of reaction times was the same for both groups of participants (group 1: A+ condition, *M* = 428 ms, *SD* = 76 ms; V+ condition, *M* = 385 ms, *SD* = 49 ms; A+V+ condition, *M* = 351 ms, *SD* = 54 ms; A−V+ condition, *M* = 372 ms, *SD* = 52 ms; A+V− condition, *M* = 456 ms, *SD* = 75 ms. Group 2: A+ condition, *M* = 454 ms, *SD* = 81 ms; V+ condition, *M* = 401 ms, *SD* = 51 ms; A+V+ condition, *M* = 352 ms, *SD* = 54 ms; A−V+ condition, *M* = 388 ms, *SD* = 54 ms; A+V− condition, *M* = 431 ms, *SD* = 71 ms; with the mean (*M*) and the standard deviation (*SD*) of ln(RTs) converted back in ms for clarity). In addition, we performed four planned comparisons to test potential interference effects in both groups, which confirmed previous results: there was no interference effect due to visual distractors, for group 1 (*t*(4) = 2.6, *p* = .06) as for group 2 (*t*(4) = 1.6, *p* = .2); there was no interference effect due to auditory distractors either, for group 1 (*t*(4) = 1.2, *p* = .3) as for group 2 (*t*(4) = 2.2, *p* = .09).

#### Test of the Race Model

The race model comparing A+V+ to V+ and A+ was significantly violated (*p*<.01) for all percentiles from the .025^th^ to .725^th^.

#### Comparison of Experiment I and Experiment II

**Figure 2:**
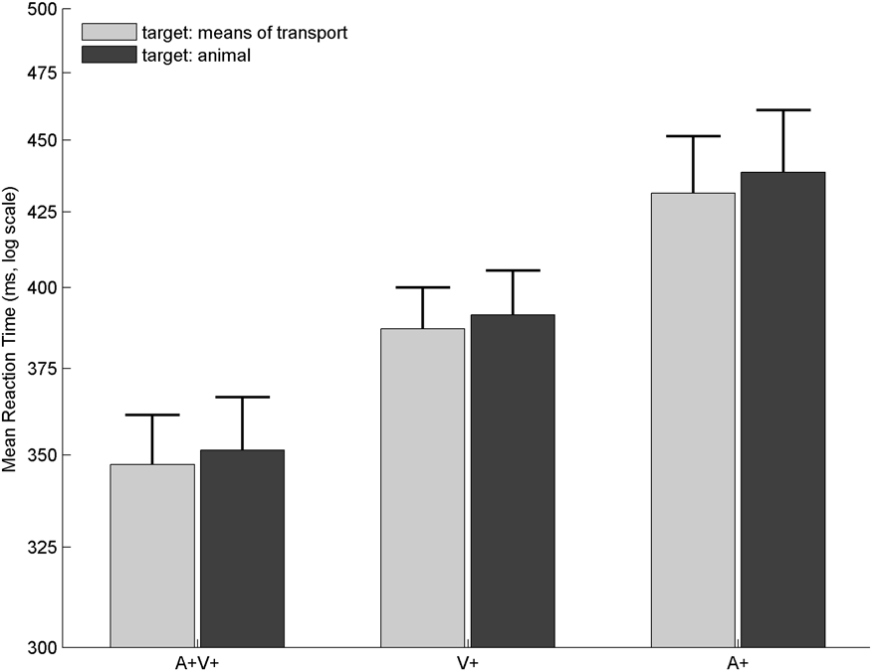
Same processing time for animals and means of transport targets. RTs for the unimodal (A+ and V+) and bimodal congruent (A+V+) conditions are presented: means of transport targets (Exp. I, light grey) are compared with animal targets (Exp. II, dark grey). RTs were first transformed to a log scale and then averaged across all participants. The log-scale was converted back to ms for displays purposes. The error bars represent one standard error of the mean. RTs to A+, V+, and A+V+ conditions were similar for animal and means of transport: there was no difference in recognition time between these two categories.

To compare the processing time of animal and means of transport as targets, we compared RTs to the A+, V+, and A+V+ conditions between the two experiments (the same participants performed the two tasks). A repeated-measures ANOVA comparing ln(RTs) with Experiment (Exp. I, Exp. II) and Condition (A+, V+, A+V+) as within-subjects factors revealed, as expected, a significant effect of the Condition (*F*(1, 11) = 114.79; *ε* = 0.9; *p*<.0001): there were differences in RTs for both experiments between the A+, V+, and A+V+ conditions. Importantly, there was no effect of the factor Experiment (*F*(1, 11) = 0.56; *p* = .5), and no effect of an interaction between Condition and Experiment (*F*(2, 22) = 0.07; *p* = .9). The speed of processing of animals and means of transport was therefore highly similar, either in the visual modality, the auditory modality, or in auditory-visual (see Table 1). In addition, it means that the different patterns of interference effects observed between the two experiments could not be explained by simple differences in the target processing time. This result is illustrated in Figure 2 (comparison of the RTs to the A+, V+, and A+V+ conditions between Experiments I and II).

### Experiment III: animals as target and distractors

#### Accuracy

The Friedman's test revealed a significant effect of Condition (V−, A−, A−V−c, and A−V−i) on the percentage of FA (χ^2^(3) = 8.41; *p*<.05). The percentage of FA was higher with a bimodal stimulus than with a unimodal one; no differences were found between the incongruent and the congruent no-go conditions (see Table 1).

There were too few misses (see Table 1) to perform a statistical analysis on these data.

#### Reaction Times

No anticipations were found and only 5 RTs were longer than 1000 ms. The distribution of the residuals of the ANOVA was not different from a normal distribution (d = 0.09; N = 60; *p*>.1).

The ANOVA comparing ln(RTs) with Condition (A+, V+, A+V+, A−V+, A+V−) as between-subject factor revealed a significant main effect of this factor (*F*(4, 44) = 19.90; *ε* = 0.6; *p*<.0001).

**Figure 3:**
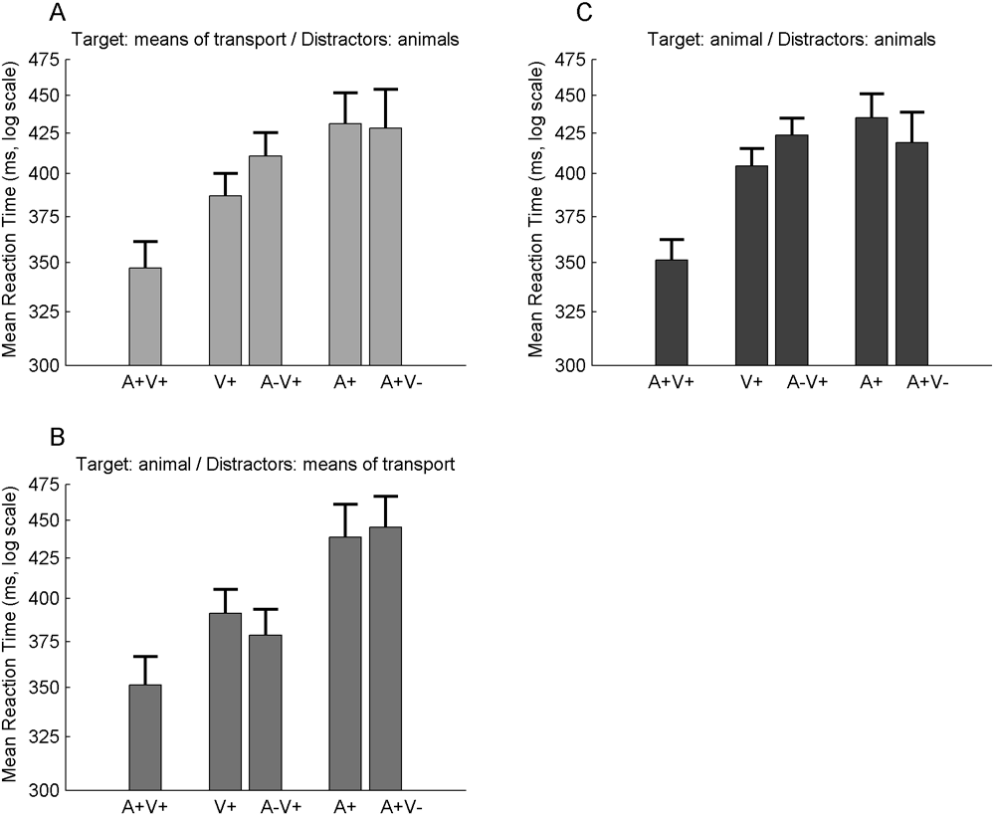
Interference effect of the animal sounds distractors. RTs of the unimodal (A+ and V+), bimodal congruent (A+V+) and bimodal incongruent (A−V+ and A+V−) conditions are presented. RTs were first transformed to a log scale and then averaged across all participants. The log-scale was converted back to ms for displays purposes. The error bars represent one standard error of the mean. Firstly, RTs to the A−V+ condition (incongruent with auditory distractors) were significantly longer than to the V+ condition *only* when auditory distractors were animal sounds (panels A and C). When the auditory distractor was not an animal, but a means of transport, there was no supplementary processing cost in presence of the auditory distractor (panel B). Secondly, RTs to the A+V− condition (incongruent with visual distractors) were always similar to RTs to A+ condition: there was no interference effect for visual distractors, whatever the category of the target or the category of the distractor (see panels A, B, and C). Finally, RTs to the bimodal A+V+ condition were clearly shorter than both unimodal A+ and V+ conditions, for the three experiments (panels A, B, and C).

The planned comparisons revealed that: (1) RTs were significantly faster for the bimodal condition (A+V+) than for the unimodal conditions (V+ and A+) considered together (*t*(4) = 14.5, *p*<.0001); (2) RTs to unimodal visual stimuli were almost similar to RTs to unimodal auditory stimuli (*t*(4) = 2.4, *p* = .03); (3) RTs to A−V+ were significantly longer than RTs to V+ (*t*(4) = 3.6, *p*<.005), thus revealing an interference effect when the animal distractor was in the auditory modality; (4) RTs to A+V− were similar to RTs to the A+ condition (*t*(4) = 1.3, *p* = .2). These results are represented in Figure 3 (panel C).

As in Experiments I and II, there were no differences between both distractors: (1) V+ was significantly different from A−V+_1_ (*t*(4) = 3.0, *p*<.01) and (2) from A−V+_2_ (*t*(4) = 3.4, *p*<.005); (3) A+ was similar to A+V−_1_ (*t*(4) = 1.1, *p* = .3) and (4) to A+V−_2_ (*t*(4) = 1.5, *p* = .2).

#### Test of the Race Model

The race model comparing A+V+ to V+ and A+ was significantly violated (*p*<.01) for percentiles from the .025^th^ to .625^th^.

## Discussion

Taken together, the main results obtained in these three experiments bring evidence that: (1) biologically relevant (animals) and non-biologically relevant objects (means of transport) are recognised within the same time, either when the comparison is done in the auditory modality alone, in the visual modality alone, or in auditory and visual modalities together; (2) when presented with a biologically relevant auditory distractor, the processing of a target object, whether it is biologically relevant or not, is disturbed.

### Same recognition time for animals and means of transport

When presented in unimodal (A+, V+) or in bimodal congruent conditions (A+V+), processing of biological or artificial categories takes the same time. The recognition of a means of transport does not take longer than the recognition of animals. This result is obtained for both modalities in isolation, as well as in bimodal situations.

In the visual modality, a similar result was put forward for the processing of categories such as faces, animals and means of transport [15], [24]. In the auditory domain, a recent study [13] reached the same conclusion, which is a similar recognition time for living versus man-made objects. However, their categories contained more disparate objects than the categories we used: living objects included animals cries and different human vocalizations (crying, sneezing, …) and man-made objects included musical instruments, bells, sirens, door closing, etc. Because of this, and/or due to the different task they employed (odd-ball paradigm), they obtained much longer RTs than in the present study (around 950 ms) suggesting that different mechanisms were involved. In addition, electrophysiological studies have also shown that the differential processing of natural and artificial categories is within the same timeframe for auditory stimuli [13] as for visual stimuli [1]. In line with previous research, our results thus confirm that, for the auditory and the visual modalities considered alone, there seems to be no specific processing for natural, biologically relevant stimuli.

Our findings also extend previous studies by considering a more natural bimodal situation. In this case, again, the same processing time was obtained in auditory-visual conditions when comparing biologically relevant and non-biologically relevant categories. In addition, a bimodal integration (as suggested by the violation of the race model) was also found for the two categories of objects. Strong associations for natural auditory-visual pairs (faces and voices) were found [17], which facilitated brain functional connectivity as indicated by fMRI. In contrast, no such associations were found for unnatural pairs (like phones and ring tones), although these non natural objects were highly familiar. The authors also found an impact of this auditory-visual association on behaviour (unimodal recognition scores). Based on this result, it could have been hypothesized that animal recognition based on auditory and visual stimuli would lead to shorter RTs than recognition of non natural objects. This hypothesis is clearly not confirmed here. This could be due to differences in the stimuli and experimental method between the two studies. In any case, our result rules out the intuitive idea that there is a clear and inevitable advantage for ecological and natural multisensory stimuli in every possible situation.

### Interference effect of the animal sounds

The second important result concerns the effect of the type of distractor on performance (RTs). We found that an interference effect was observed only for auditory distractors, and only when the distractor was an animal sound (whatever the animal). Animal sounds seem to be able to disrupt the visual processing of a target, whether the target's category is an animal or a means of transport.

The fact that we observed the same baseline processing time for animals and means of transport is important for the interpretation of this result. It implies that the specific attentional processing observed for animal sounds can not be explained by simple differences in target processing. This result also suggests that the two categories of objects used here were of the same degree of complexity.

For visual distractors as well as for non-biologically relevant auditory distractors, no increase in the concurrent target processing time was observed. This result suggests, in these cases, that a parallel processing of target and distractors objects was achieved, or that the distractors were effectively ignored. In contrast, when the distractor was auditory and biologically-relevant, such parallel processing or efficient suppression of the distractor could not be achieved. The interference effect by an irrelevant animal sound that we demonstrate thus brings new evidence on the vulnerability of attentional selectivity across sensory modalities. Brain mechanisms able to disrupt attention and to detect irrelevant but potentially important distractors are crucial from an adaptative perspective. It seems plausible that, considering that audition is an efficient warning sense, the processing of a biologically relevant stimulus presented in this modality is mandatory, whatever the current state of attention. This specific effect might also reflect a parallel processing of biologically meaningful and potentially fear-relevant information [25], possibly involving a subcortical pathway bypassing the auditory cortex. The visual processing would then be slowed down to concentrate available capacities to process the potentially relevant information.

Previous results on the effect of auditory distractors on visual performance are not fully consistent with each other. A first study [26] found that semantically incongruent bimodal targets produced the same RTs as the corresponding unimodal targets. In contrast, with linguistic-type stimuli, another study [27] did find a significant interference effect between the incongruent and unimodal conditions, with longer RTs to the incongruent conditions. Finally, recently, a stronger effect of a visual distractor on auditory recognition than of an auditory distractor on visual recognition was showed [28]. They interpreted this result as visual dominance in object recognition. One major difference between this latter study and the present one concerns the direction of attention. In the present study, the attention was divided between the two modalities. The participant did not focus their attention on one modality, as task-relevant information could appear in auditory or visual form in any given trial. Instead, participants focused on a particular object. In the latter study [28] the attention was directed to a single modality (either auditory or visual depending on the blocks). More generally, the difference in interference effects observed in all these studies might be explained by differences in the semantic category of the stimuli, their realism, and the involved attentional parameters.

A further point that is worth investigating is whether the observed interference can be explained by some low-level features specific to animal sounds. Interestingly, the interference effect was found to be similar for different animal sounds. It could be because processing animal sounds entails computing some statistical regularities that characterize the physics of the source of the sound (see [29]–[33]). Studying the interplay between low-level features and semantic category interference effects might give an important insight into the organization of our stored knowledge of object concepts.
